# Subpubic Cartilaginous Pseudocyst: Orthopedic Feature with Urological Consequences

**DOI:** 10.1155/2014/176089

**Published:** 2014-01-16

**Authors:** Fawzy Farag, Ingrid van der Geest, Christina Hulsbergen-van de Kaa, John Heesakkers

**Affiliations:** ^1^Department of Urology, Radboud University Medical Centre, P.O. Box 9101, 6500 HB Nijmegen, The Netherlands; ^2^Department of Urology, Sohag University Hospitals, Egypt; ^3^Department of Orthopedic Surgery, Radboud University Medical Centre, The Netherlands; ^4^Department of Pathology, Radboud University Medical Centre, The Netherlands

## Abstract

*Introduction*. Masses arising from structures adjacent to the female urethra can cause obstructive urinary symptoms. Subpubic cartilaginous pseudocyst is a rare degenerative lesion of pubic symphysis that may cause these symptoms. *Materials and Methods*. A 61-year- and 57-year-old women presented with symptoms of difficult micturition and dyspareunia. Physical examination revealed a painless smooth, rounded, firm, and cystic mass, at the anterior vaginal wall of about 4 cm width. The mass caused inward deviation of the external urethral meatus. Cystoscopy and MRI were done. *Results*. Cystoscopy of case 1 (61 y) demonstrated anterior external urethral compression with normal urethral mucosa. Cystoscopy was not possible in case 2 (57 y) because the urethra could not be entered under local anesthesia. MRI showed almost the same findings in both cases: midline, rounded, and cystic mass ~3 × 3 × 4 cm, anterosuperior to the urethra, and posteroinferior to the pubic symphysis, with normal features of the urinary bladder. Open surgical excision of theses lesions was performed in both patients. Histopathologic assessment of the specimen obtained from both patients showed degenerated hyaline with areas of fibrinous and mucoid degeneration, a picture suggestive of cartilaginous subpubic pseudocyst. After 11-month and 4-month followup of patients numbers 1 and 2, respectively, there is no evidence of local recurrence of the lesion, either clinically or radiologically and both patients void empty. *Conclusions*. Subpubic cartilaginous pseudocysts are rare benign lesions with only 13 cases were reported in the literature. Patients present with a spectrum of gynecological and/or urological manifestations. Sizable lesions severely compressing the urethra need surgical excision to restore the voiding function.

## 1. Introduction

The bladder outlet obstruction (BOO) is one of the voiding dysfunctions that presents with a spectrum of symptoms of hesitancy, slow or intermittent urinary stream, straining, and terminal dribble [1]. BOO is uncommon in women. Urethral stenosis, urethral- or periurethral masses, and obstructing vaginal tapes can be leading causes of these symptoms in women. Masses arising from adjacent structures to the female urethra, for example, the pubic symphysis, can be expected to cause similar complaints.

The pubic symphysis is a midline joint connecting the pubic bones of the pelvis, with an intervening fibrocartilaginous disc. It can resist tension and shearing; however, it has the ability to widen during pregnancy [2].

Subpubic cartilaginous pseudocyst is a rare degenerative lesion related to the pubic symphysis. It was first described by Algucial-Garcia and Littman [3] in the year 1996. The authors reported the occurrence of this lesion in two postmenopausal women. A few more cases have been reported since then, most of them occurred in multiparous women who presented with a variety of urological and/or gynecological manifestations [4]. In this paper, we report our centre's experience with two new cases of subpubic cartilaginous pseudocyst.

## 2. Case Presentation

### 2.1. Case 1

**Figure 1:**
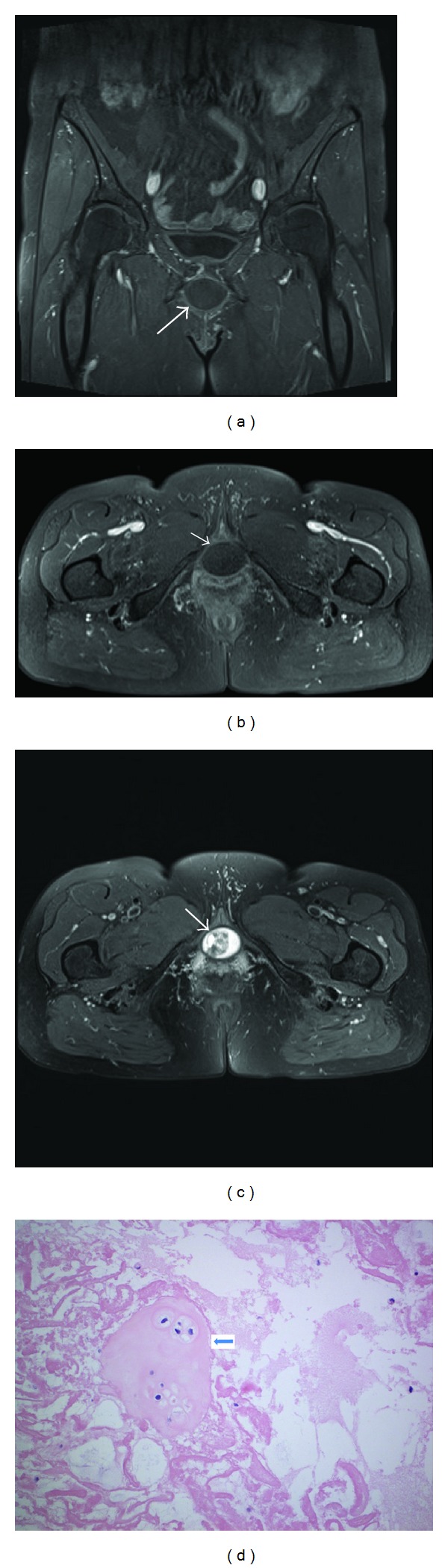
Case 1. A 61-year-old postmenopausal lady presented with voiding dysfunction. Examination revealed painless smooth, rounded, firm, and cystic mass, at the anterior vaginal wall of about 4 cm width related to the pubic symphysis. (a) Coronal T1-weighted image of a rounded, hypointense pseudocyst compared to the muscle tissue. (b) Axial T1-weighted image of the pseudocyst. (c) Axial T2-weighted image depicting a hyperintense, heterogenous pseudocyst. (d) The histopathologic features of the cyst. The 200x image shows an island of hyaline cartilage (arrow) floating in a degenerated fibrinous and mucoid substance.

A 61-year-old, postmenopausal, lady, G 3, P 3, with normal vaginal deliveries, presented with complaints of difficult micturition and dyspareunia. On physical examination, a painless, smooth, rounded, firm, and cystic mass was observed at the anterior vaginal wall of about 4 cm width; it appeared to be related to the pubic symphysis. The mass caused inward deviation of the external urethral meatus. Cystoscopy revealed compressed external urethral meatus with normal urethral mucosa. Urodynamic data are not available for this patient; however the history and physical examination were suggestive of BOO. Magnetic resonance imaging (MRI) showed a midline, rounded, cystic mass, of 3.2 × 3.0 × 3.9 cm dimensions, located anterosuperior to the urethra and posteroinferior to the pubic symphysis (Figures 1(a)–1(c)). There were no abnormalities of the surrounding pelvic structures, an image suggestive of benign subpubic cartilaginous pseudocyst.

Surgical excision of the mass was done under spinal anesthesia. A transurethral catheter was inserted and a suprapubic horizontal incision was made. A blunt dissection was made ventrally of the pubic bone down to the cyst. When the cyst was opened it showed clear liquid content. The cyst was then sharply dissected-off of the pubic ramus with scissors and curettage. The vaginal wall and urethra were intact and felt separate from the cyst. The periosteum of the pubic symphysis was reapproximated with vicryl.

Histopathologic examination of the specimen showed degenerated hyaline with areas of fibrinous and mucoid degeneration, a picture suggestive of cartilaginous subpubic pseudocyst (Figure 1(d)). Thirteen days after surgery, the patient came to the emergency unit with complaint of fever, pain, and discharge at site of incision due to local wound infection. Rinsing of the wound was done and the condition was resolved within 3 weeks (grade II Clavien Classification of Surgical Complications). After 11 month of followup, there was no clinical or radiological evidence of local recurrence of the lesion. The patient has no voiding difficulties anymore.

### 2.2. Case 2

A 56-year-old postmenopausal lady, G1 P1, with normal vaginal delivery, presented with complaint of difficult micturition in sitting position; she had to lean backward to initiate and to maintain the act of voiding. On physical examination, a painless, smooth, rounded, firm, and cystic mass was noticed at the anterior vaginal wall, of 4 cm width, which appeared to be related to the pubic symphysis. The mass caused inward deviation of the external urethral meatus.

Urodynamics investigation revealed a bladder capacity of 243 cc, evidence of detrusor overactivity, postvoid residual urine of 120 cc, and Pdet. Qmax of 21 cm H_2_O. Cystoscopy was not possible due to severely compressed, posteriorly deviated urethra.

**Figure 2:**
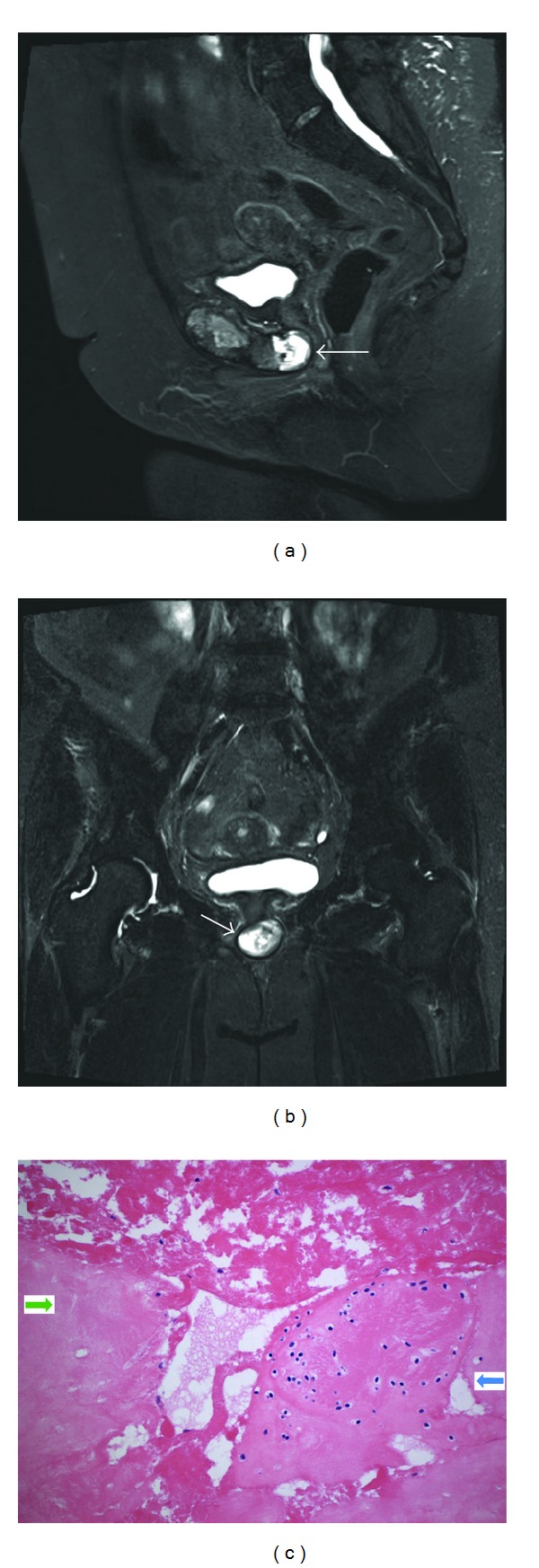
Case 2. A 56-year-old, postmenopausal, lady, presented with voiding dysfunction. Examination revealed painless smooth, rounded, firm, and cystic mass, at the anterior vaginal wall of about 4 cm width related to the pubic symphysis. (a) Sagittal T2-weighted image of a well delineated hyperintense pseudocyst, related to the pubic symphysis. (b) Coronal T2-weighted image of the pseudocyst. (c) The histopathologic features of the cyst. The 200x image shows an area of vital hyaline cartilage (blue arrow) with fibrinous degeneration of its centre. The cartilage is surrounded by partly cystic fibrous substance. The green arrow points to part of a necrotic cartilage.

MRI showed a midline, cystic mass that measured about 3.0 × 3.8 × 2.7 cm, located anterosuperior to the urethra and posteroinferior to the pubic symphysis, with a wall thickness of 9 mm. (Figures 2(a) and 2(b)).

**Figure 3:**
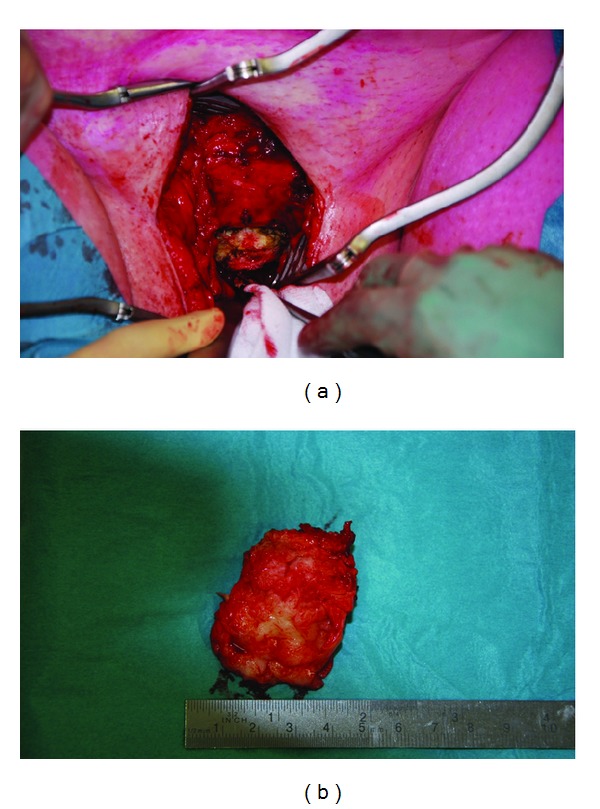
Case 2. A 56-year-old postmenopausal lady presented with voiding dysfunction. (a) Operative picture of smooth, rounded, firm, and cystic mass, at the anterior vaginal wall of about 4 cm width connected to the pubic symphysis. (b) Postoperative picture of the excised pseudocyst.

Surgical excision of the mass was done under general anesthesia. A urethral catheter was inserted. Through a Pfannenstiel incision, sharp dissection was done with scissors deep until the level of the cyst was reached (Figures 3(a) and 3(b)). The bladder and urethra were not connected to the cyst. The cyst was opened; it showed a solid whitish tissue content with no fluid or pus. The cyst was dissected all around using sharp scissors, diathermy, and finally with curettage. Hemostasis was done and low vacuum drain was inserted.

Histopathologic assessment of the specimen revealed a degenerated hyaline with areas of fibrinous and mucoid degeneration, a picture suggestive of cartilaginous subpubic pseudocyst (Figure 2(c)). Five days later, the patient came to the emergency unit with discharge at the site of the wound due to infection. Rinsing of the wound was done, and then the condition was resolved within 4 weeks. The patient developed a hematoma at the site of operation with reduced hemoglobin which necessitated 2 units of blood transfusion (grade II Clavien Classification of Surgical Complications). The patient has no voiding difficulties anymore and can void empty for the last 4 months after the date of surgery.

## 3. Discussion

The subpubic cartilaginous pseudocyst is a rare degenerative lesion of the pubic symphysis; it is usually associated with narrowing and subchondral sclerosis of the pubic symphyseal joint. The fibrocartilaginous disc of the pubic symphysis joint may undergo mucinous cystic changes as a result of pregnancy, vaginal delivery, or pelvic trauma [3]. The mucinous and chondrocytic elements of the subpubic or parapubic cartilaginous pseudocyst give the lesion its radiologic features [4]. However, Tan et al. [5] believe that a parasymphyseal pubic cartilaginous cyst may have radiological features that may mimic a pelvic girdle chondrosarcoma. Therefore, they suggested taking a biopsy for histopathologic work-up with regular followup imaging of the lesion to avoid unnecessary surgical interference.

A comprehensive search of the literature revealed that a total of 11 cases [3–12] of similar conditions were reported before. The lesion was observed in nine women, 8 of them were multiparous, and in 1 man [7] who had a gas containing cyst on MRI. The lesions were described either as sub- or parapubic cartilaginous cysts. The complaints of the patients were either painful or painless vulvar mass, dyspareunia, or obstructive voiding symptoms up to acute urinary retention. Most of these patients underwent surgical excision of their cysts, although some other patients [5, 8] were conservatively treated with followup MRI to monitor any change in the size or criteria of their cysts.

Clinical assessment of our two patients revealed the presence of the midline, hard, cystic lesion that caused deviation of the urethral external meatus downward and backward, to an extent that urethroscopy was not possible in case number 2. Urodynamic evaluation of patient number 2 revealed an obstructive pattern which was taken into account when we made a final decision of surgical interference. This test was not done in most of the previous cases reported in the literature and we think it should be applied together with urethroscopy as routine investigations in patients with suspicious subpubic cartilaginous pseudocysts. In the two patients, the masses were sizable enough to compress the urethra and disturb the act of voiding which finally necessitated surgical removal of these masses.

The histopathologic patterns of the masses in both cases were almost the same being a composite of cartilaginous wall with intersections of connective tissue showing cystic degenerative changes and necrosis. This also goes in line with the histopathologic patterns of the previously reported cases in the literature which supports the assumption that these lesions develop as a result of degenerative changes of the fibrocartilaginous disc of the pubic symphyseal joint that may undergo mucinous cystic changes [3].

## 4. Conclusions

Subpubic (or parapubic) cartilaginous pseudocysts are rare lesions with only 13 cases being reported in the literature since the year 1996 till now. The patients present with a spectrum of gynecological and/or urological manifestations. Characteristic imaging features of these lesions are being developed which might save a number of unneeded biopsies and even unnecessary surgical excision of these benign lesion. However, in some patients with sizable lesions, the urethra could be severely compressed, and surgical excision should be considered in order to restore the voiding function in these patients.
